# Ambition and extreme behavior: relative deprivation leads ambitious individuals to self-sacrifice

**DOI:** 10.3389/fpsyg.2023.1108006

**Published:** 2023-07-12

**Authors:** Elena Resta, Molly Ellenberg, Arie W. Kruglanski, Antonio Pierro

**Affiliations:** ^1^Department of Developmental and Social Psychology, Sapienza University of Rome, Rome, Italy; ^2^UniSR-Social.Lab, Vita-Salute San Raffaele University, Milan, Italy; ^3^Department of Psychology, University of Maryland, College Park, MD, United States

**Keywords:** ambition, extreme behavior, relative deprivation, quest for significance, justice sensitivity, self-sacrifice

## Abstract

Ambitious people are characterized by strong motivation toward great and valuable objectives, with the superordinate goal to gain respect and recognition from others. Recent literature regarding ambition demonstrated that it leads individuals to engage in extreme behavior. However, no previous research has investigated under which conditions the relation between ambition and extremism is enhanced. Across two studies, we tested the hypothesis that ambitious individuals are more prone to engage in extreme behavior in the face of relative deprivation (i.e., justice sensitivity), than their less ambitious counterparts. We confirmed our predictions employing a cross-sectional design with an American sample (Study 1) and an experimental design with an Italian sample (Study 2). The present research adds theoretical knowledge and empirical support to the existing literature on ambition, extreme behavior, and relative deprivation, and provides fruitful insight into strategies for preventing extremism.

## 1. Introduction

In Kruglanski et al. (2021) conceptualization, extreme behaviors are “the results of motivational imbalance” (p. 265). Humans typically find themselves in a state of motivational balance, in which all of their basic needs are satisfied. However, it may happen that one need becomes predominant over other basic needs, generating a state of motivational imbalance. Such imbalance implies that behaviors devoted to satisfaction of the dominant need are enacted, leading to the sacrifice of other needs and thus to extreme behavior. When one need dominates over other needs, individuals devote their energies, attention, thoughts, and actions toward the satisfaction of the prioritized need. Consequently, people become emotionally dependent on the developments in the dominant need fulfillment and neglect other needs. Such negligence, if prolonged over time, becomes damaging. In this vein, Burke (1999) showed that individuals reporting greater workaholism were less satisfied in extra-work areas such as family, friends, and community. This result is consistent with research showing that workaholic individuals tend to sacrifice financial, health and social goals in favor of their work (e.g., Machlowitz, 1980; Bonebright et al., 2000; Robinson et al., 2001; Ng et al., 2007). Usually, extreme behaviors are referred to antisocial actions but, as demonstrated by Dugas et al. (2016), extreme behaviors can also be prosocial.

Literature has identified the quest for significance, the fundamental need to matter, have dignity, and merit respect (Kruglanski et al., 2022), as the principal antecedent of extremism. Indeed, people who engage in extreme behavior typically do so in order to gain prestige and recognition (Kruglanski and Bertelsen, 2020). More recently, Resta et al. (2022a) demonstrated that ambition, conceived as a manifestation of the quest for significance, can result in extreme behavior. Ambitious individuals are characterized by strong persistence in pursuing valuable goals, with the superordinate goal to gain respect and recognition from others, up to the point that they engage in extreme behavior (Resta et al., 2022a,b). Due to this constant search for meaning, ambitious individuals are particularly sensitive to situations in which their worth and respect (i.e., significance) are dishonored (Resta et al., 2022c). Our aim is to investigate if ambitious individuals are more prone to engage in extreme behavior when their significance is challenged, for instance under conditions of personal injustice, such as that of relative deprivation.

### 1.1. Relative deprivation

Every day, individuals perceive that they are treated unfairly or unjustly. We may perceive that other people have been given benefits which we have been denied, or that others are given opportunities or rewards to which we were entitled or of which we were more deserving. This perception is often explained within the context of relative deprivation (Stouffer et al., 1949), in which individuals make comparisons between themselves and relevant reference groups and perceive that they have been unjustly disadvantaged in comparison to that reference group, thus engendering feelings of frustration, anger, and resentment (e.g., Crosby, 1976). In response to such negative feelings, an individual experiencing relative deprivation will pursue any actions they believe will improve their situation, including those which solidify their social identity and aim to raise the status of their social group (e.g., Ellemers, 2002). Indeed, relative deprivation has been found to predict a range of actions, from international migration (Stark and Taylor, 1991) to preference for immediate gratification and gambling urges (Callan et al., 2011). Cross-cultural evidence for relative deprivation as a phenomenon and predictor of poor subjective wellbeing and political violence has been found among participants in Japan (Ohno et al., 2023), Brazil, Turkey, Belgium, France (Adam-Troian et al., 2020), the Netherlands, and Singapore (van den Bos et al., 2015). Furthermore, Grant (2008) demonstrated that skilled immigrants in the Canadian labor market who experienced relative deprivation reacted with strong protest intentions and engagement in protest actions. Similarly, Stiles et al. (2000) found in a sample of adolescents that economic deprivation induced negative feelings which, in turn, predicted adoption of deviant behavior such as violence and drug use. Moreover, Zoogah (2010) showed that employees who felt relatively deprived were more prone to participate in development activities in order to redress perceived disadvantages. More recently, Schreurs et al. (2021) found that employees who perceived themselves as overqualified for their jobs were more likely to experience relative deprivation – that colleagues were being treated better than they were – which in turn predicted self-reported counterproductive and unethical behavior at work. Interestingly, this relationship was stronger among more ambitious employees, whose sense of relative deprivation when comparing their own treatment to that of their coworkers was amplified. Additionally, group relative deprivation, specifically, a perception that immigrants were treated better by the government than one’s own group, was found to completely explain the negative relationship between socioeconomic status and perceived threat from immigrants among Europeans (Meuleman et al., 2020).

### 1.2. Ambition, the quest for significance, and extreme behavior

Given ambitious people’s high need to stive for their significance up to the point to engagement in extremism, it is reasonable to hypothesize that ambitious individuals might be especially affected by relative deprivation. In fact, relative deprivation concerns situations in which people’s worthiness is disrespected. Being surpassed by others in an unfair way or seeing others obtaining what you deserve can be recognized as situations where one perceives a reduction in personal significance. The present research aims to explore what happens when ambitious individuals are hindered, disadvantaged, or given fewer opportunities in the process of reaching their significance. We predict that when personal significance is challenged by conditions of personal injustice, individuals, especially ambitious ones, increase their willingness to self-sacrifice for a valued objective in order to restore their dignity and respect.

Literature on significance quest suggests that even temporary experiences of humiliation or failure can drive individuals to the engagement in extreme actions to restore their lost significance (Kruglanski et al., 2021, 2022). In this regard, research showed that when individuals feel humiliated, they are prone to support their political orientation more strongly (Webber et al., 2018). Dugas et al. (2016) found that people who feel socially rejected or have failed are more willing to self-sacrifice. Moreover, Bélanger (2013) reported that members of Liberation Tigers of Tamil Eelam (an extremist group) who felt insignificant were more prone to support extreme behavior, such as political violence. Similarly, religious people experiencing sexual guilt (i.e., significance loss) were found to be more prone to self-sacrifice (Belanger et al., 2019).

### 1.3. The present research

Building off of the previous research demonstrating that ambitious people, characterized by higher levels of significance quest, are more sensitive to personal injustice (Resta et al., 2022b), we presently hypothesized that relative deprivation can moderate the relationship between ambition and extreme behavior. Specifically, as previously noted, ambitious individuals put their quest for significance above other motives, and are therefore prone to make sacrifices for their quest for significance. Therefore, we predict that ambitious individuals, when confronted with relative deprivation (vs. everyday life situations), are more prone to engage in extreme behavior to restore their significance. We tested our predictions across two studies. In Study 1, through a cross-sectional design and employing an American sample, we hypothesized that ambition would lead to more extreme behavior under conditions of strong relative deprivation. In Study 2, we tested the same hypothesis though an experimental design and employing an Italian sample. In both studies we used the Justice Sensitivity Scale (Schmitt et al., 2010) to assess and manipulate the relative deprivation. This measure is designed to capture feelings of frustration, anger, and resentment, as they are the emotional correlates of relative deprivation. It is noteworthy that, given ambitious individuals’ strong need for significance, justice sensitivity might mediate the relationship between ambition and extreme behavior. Indeed, one can argue that a strong quest for significance could make individuals (i.e., the ambitious ones) more sensitive to situations in which their significance is threatened, which in turn would lead to extreme behavior. However, in the present study we focused on the moderating role of justice sensitivity because we were interested in adding literature regarding the *conditions* in which ambitious individuals are prone to engage in extreme behavior. The above studies were approved by the Ethics Committee of the Department of Developmental and Social Psychology at Sapienza University of Rome (protocol 808). Materials employed in both studies can be found in Supplementary material.

## 2. Study 1

### 2.1. Method

#### 2.1.1. Procedures, design, and participants

To determine the minimum sample size, we conducted an *a priori* power analysis using G*Power 3.1.9.4 (Faul et al., 2009). Assuming small to medium effect size (*f*^2^ = 0.04), with three predictors, power set to 0.80 and α set to 0.05, the analysis revealed a required sample size of 277. We recruited 299 American adults (55.9 percent male), aged 18 to 50 years old (*M* = 31.30, *SD* = 8.48), to take part in a cross-sectional study. Among participants, 33.4 percent had a high school degree or equivalent, 32.1 percent had earned a bachelor’s degree, 27.1 percent had earned a master’s degree, and 4.7 percent had earned a PhD. Participants were enrolled online through a paid procedure provided by Prolific. After providing informed consent, participants were asked to fill out an online questionnaire aimed to assess justice sensitivity, ambition, and extreme behavior.

### 2.2. Measure

#### 2.2.1. Justice sensitivity

Participants’ justice sensitivity was assessed through an abridged and adjusted version of the Justice Sensitivity Scale’s victim perspective (Schmitt et al., 2010). Individuals were asked to indicate their agreement with statements regarding feelings of frustration and discomfort in response to situations of relative deprivation (five items, e.g., “*I am bothered when people get what I deserve*”). Responses were provided on a five-point Likert scale (1 = “*Definitely disagree*”; 5 = “*Definitely agree*”) and were averaged to form a single justice sensitivity score (Cronbach’s alpha = 0.82).

#### 2.2.2. Ambition

Participants’ ambition was assessed through the English version of the Ambition Scale (Resta et al., 2022b). Individuals were asked to indicate their agreement with statements regarding aiming at great, valuable objectives, and striving for success, recognition, and respect from others (ten items, e.g., “*Attaining recognition, respect, and consideration for what I do is very important to me*”). Responses were provided on a five-point Likert scale (1 = “*Definitely disagree*”; 5 = “*Definitely agree*”) and were averaged to form a single ambition score (Cronbach’s alpha = 0.91).

#### 2.2.3. Extreme behavior

Participants’ extreme behavior was assessed through an abridged version of the Self-Sacrifice Scale (Bélanger et al., 2014). First, participants were asked to list a cause they considered personally important. Subsequently, they responded to five items designed to evaluate how much they would sacrifice their relations with relatives, their money, or themselves, for the sake of the cause they listed (e.g., “*I would defend a cause to which I am truly committed even if my loved ones rejected me*”). Responses were provided on a seven-point Likert scale (1 = “*Do not agree at all*”; 7 = “*Very strongly agree*”) and were averaged to form a single extreme behavior score (Cronbach’s alpha = 0.72).

### 2.3. Results

Preliminary analyses revealed a positive and significant correlation between justice sensitivity and ambition (*r* = 0.14, *p* = 0.015), as well as between extreme behavior and ambition (*r* = 0.18, *p* = 0.001), consistent with the previously cited literature regarding characteristics of ambitious individuals and their proneness to engage in extremism (Resta et al., 2022a,c). Otherwise, justice sensitivity was not related to extreme behavior (*r* = 0.09, *p* = 0.141).

**Figure 1:**
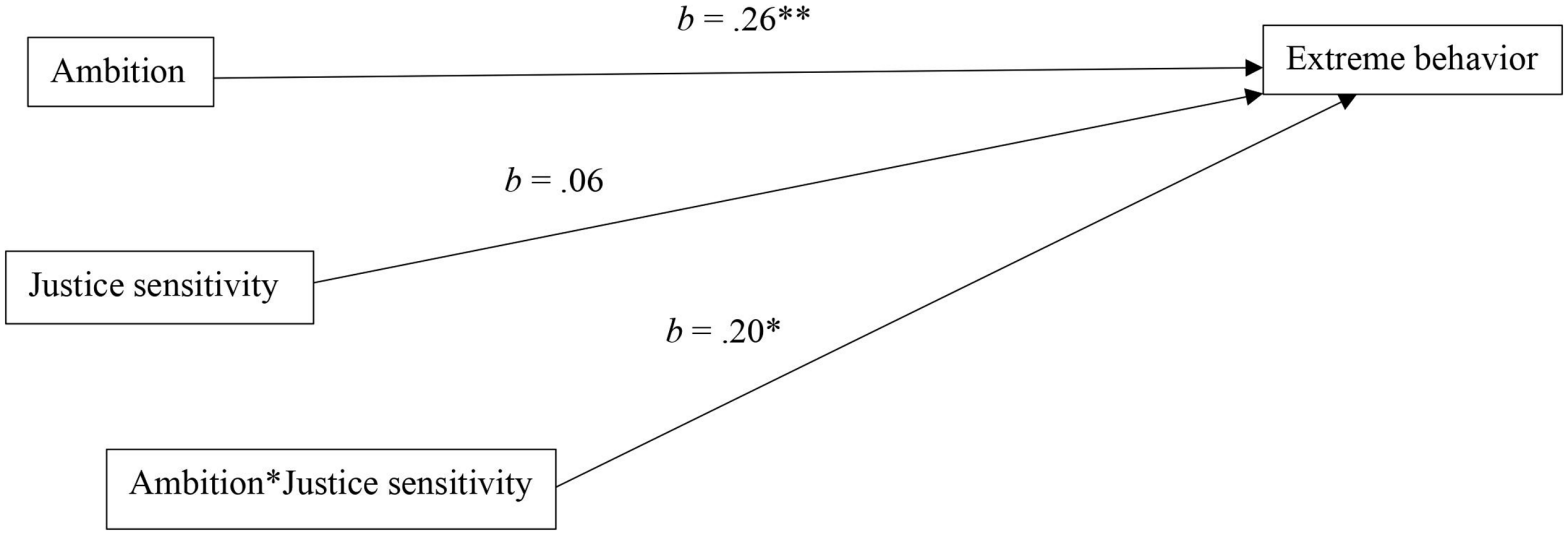
Results of the moderated model tested in Study 1 (*N* = 299). *p < 0.05 **p < 0.01.

To test the moderating role of justice sensitivity in the relationship between ambition and extreme behavior, we used PROCESS v3.5 (Hayes, 2018), Model 1. As shown in Figure 1, ambition positively and significantly predicted extreme behavior [*b* = 0.26, (95% CI = 0.10, 0.42), *SE* = 0.083, *t* = 3.139, *p* = 0.002], whereas justice sensitivity did not predict extreme behavior [*b* = 0.06, (95% CI = −0.10, 0.21), *SE* = 0.079, *t* = 0.747, *p* = 0.456]. Importantly, we found a positive and significant interaction between ambition and justice sensitivity on extreme behavior [*b* = 0.20, (95% CI = 0.04, 0.37), *SE* = 0.083, *t* = 2.445, *p* = 0.015], indicating that the positive relationship between ambition and extreme behavior was stronger for higher scorers on justice sensitivity.^1^

**Figure 2:**
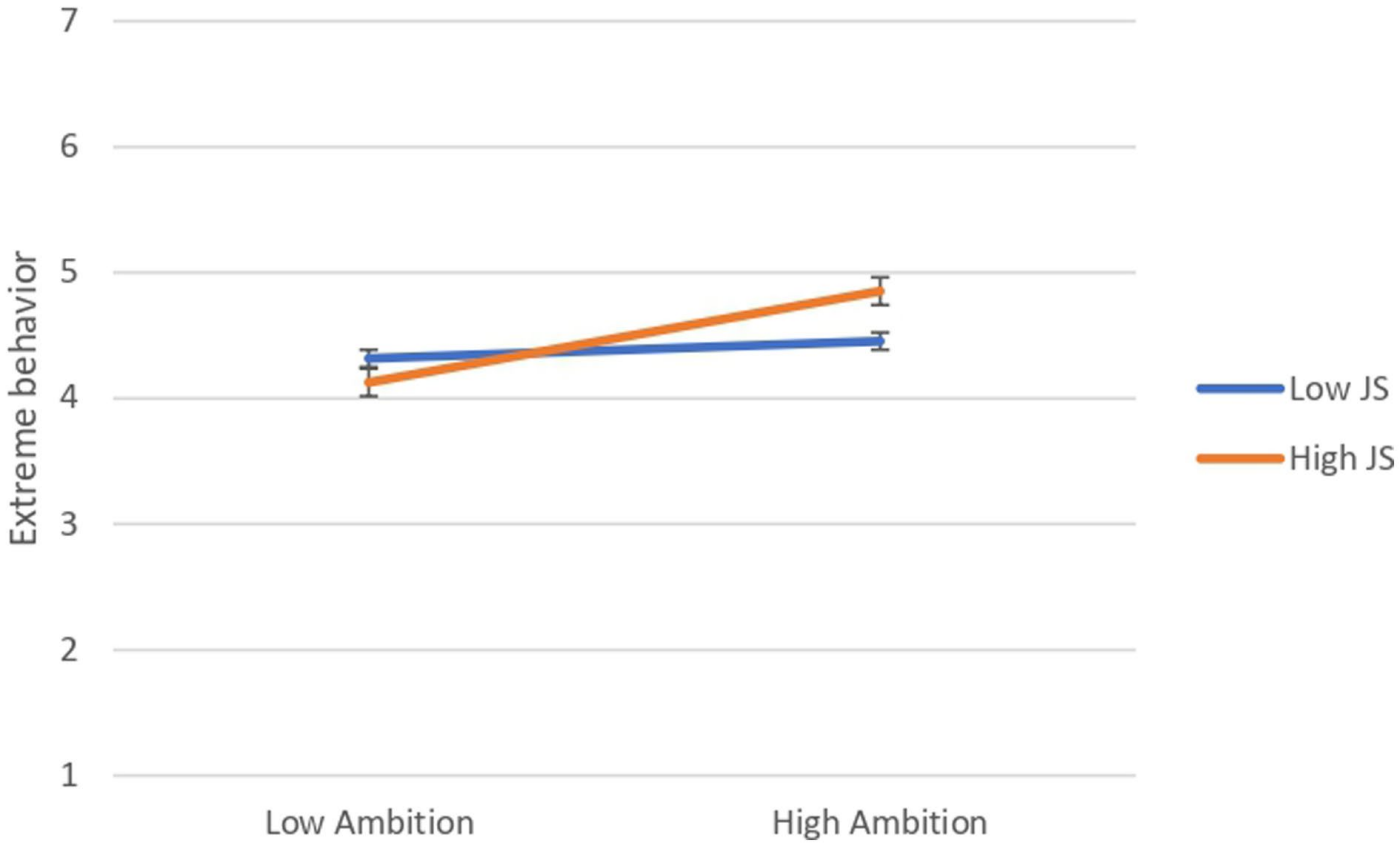
Results of simple-slope analysis to interpret the effect of the interaction between ambition and justice sensitivity on extreme behavior (Study 1; *N* = 299). N.B. JS = Justice sensitivity.

A simple-slope analysis revealed that the relationship between ambition and extreme behavior was positive and significant at high levels of justice sensitivity (1 *SD* above the mean), [*b* = 0.44, (95% CI = 0.22, 0.66), *SE* = 0.112, *t* = 3.916, *p* < 0.001], whereas this relation was non-significant at low levels of justice sensitivity (1 *SD* below the mean), [*b* = 0.08, (95% CI = −0.13, 0.30), *SE* = 0.109, *t* = 0.761, *p* = 0.447], (see Figure 2).

### 2.4. Discussion

The results of Study 1 constitute the first evidence of the moderating role of relative deprivation in the relationship between ambition and extreme behavior. Specifically, we found that ambitious individuals are more prone to engage in extreme behavior when they have high levels of justice sensitivity. Otherwise, ambitious people were not more likely to engage in extreme behaviors when they had low levels of justice sensitivity. However, the findings of Study 1 were obtained through correlational data which prevented us from drawing firm conclusions. In particular, Study 1 does not provide information as to whether ambitious individuals, when confronted with induced feelings of relative deprivation, increase their extreme behavior. Additionally, the present study was conducted with an American sample, precluding generalization of the findings to other populations. To address those issues, we implemented a second study.

## 3. Study 2

To find further support for and generalize the results of Study 1, we conducted a second study, using a sample of Italian participants. We tested the same hypothesis of Study 1, this time through an experimental design in which we manipulated justice sensitivity.

### 3.1. Method

#### 3.1.1. Procedures, design, and participants

To determine the minimum sample size, we conducted an *a priori* power analysis using G*Power 3.1.9.4 (Faul et al., 2009). Assuming medium effect size (*f* = 0.25), power set to 0.80 and α set to 0.05, the analysis revealed a required sample size of 180. We recruited 200 Italian adults to take part in an experimental design in which we manipulated justice sensitivity through a recall task. Those who did not respond to the manipulation were excluded. Thus, the final sample was constituted of 193 participants (51.3 percent female), aged 19 to 49 years old (*M* = 27.45, *SD* = 7.18). Among the participants, 43.5 percent had a high school degree, 51.3 percent had earned a bachelor’s degree, and 5.2 percent had earned a PhD. Participants were recruited online through a paid procedure provided by Prolific. After giving informed consent, participants were asked to fill out an online questionnaire, the first part of which was designed to assess ambition. Subsequently, participants were randomly assigned to one of two possible conditions: (1) justice sensitivity, or (2) control. Finally, extreme behavior was measured.

### 3.2. Measure

#### 3.2.1. Ambition

Participants’ ambition was assessed through the Italian version of the Ambition Scale (Resta et al., 2022b), the same scale employed in Study 1 (Cronbach’s alpha = 0.89).

#### 3.2.2. Manipulation of justice sensitivity

Participants were randomly assigned to one of two conditions. In both, individuals were asked to recall three situations. In the “justice sensitivity” condition, participants were first asked to describe a time when they felt bothered because they had fewer opportunities than others to fulfill their ambitions. Second, they were asked to carefully describe a time when they felt frustrated because they had to work hard to achieve a goal that others easily gained. Finally, they were asked to carefully describe a situation where they felt bothered because others got what the participants believed they themselves deserved. In the “control” condition, participants were first asked to describe what constituted their last meal. Then, they were asked to carefully describe the prototypical restaurant they are used to going to. Last, they were asked to describe their typical day.

#### 3.2.3. Manipulation check

To verify whether our manipulation worked, after the recall task, all participants indicated *via* a single item the extent to which they felt unjustly treated in the situations about which they had just written. Responses were provided on a 10-point Likert scale (1 = “*Not at all*”; 10 = “*A lot*”). We expected that the participants assigned to the “justice sensitivity” condition would score higher on this measure than those assigned to the “control” condition.

#### 3.2.4. Extreme behavior

After the manipulation check, we assessed extreme behavior in all participants through the same scale employed in Study 1. However, in this study, participants were asked to list one objective (instead of a cause) that was very dear to them and subsequently, they responded to items designed to evaluate the extent of sacrifice *in the present moment*, rather than in general (e.g., “*Right now, I would strive to achieve this objective, even if my loved ones rejected me*”). Responses were averaged to form a single extreme behavior score (Cronbach’s alpha = 0.73).

**Figure 3:**
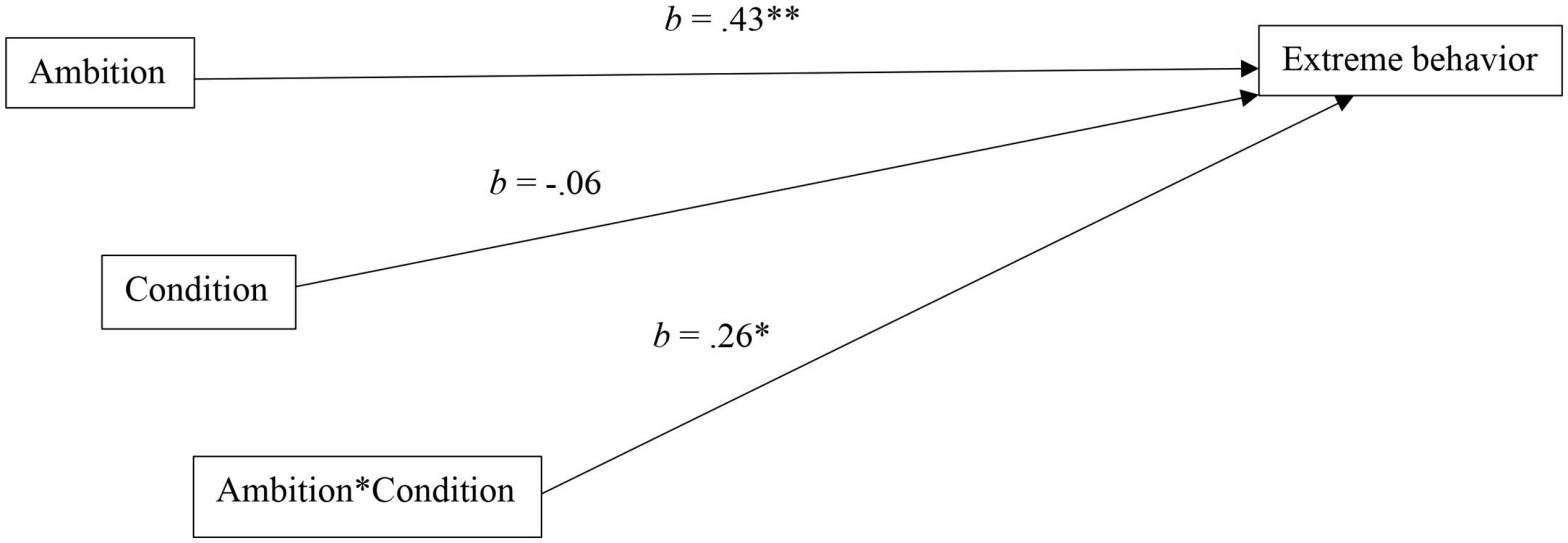
Results of the moderated model tested in Study 2 (*N* = 193). *p < 0.05 **p < 0.01.

### 3.3. Results

Preliminary analyses confirmed the results of Study 1, showing a positive and significant correlation between ambition and extreme behavior (*r* = 0.22, *p* = 0.002) and a weaker, albeit nonetheless positive and significant correlation between ambition and condition (*r* = 0.16, *p* = 0.001). Given that condition was randomly assigned after ambition had already been measured, it is likely that this weak correlation arose arbitrarily. Similarly to Study 1, the assignment to the justice sensitivity (vs. control) condition was not related to extreme behavior (*r* = −0.01, *p* = 0.843).

To test if the exposure to manipulation (vs. control) generated higher scores on perception of injustice, we performed an independent sample *t*-test. Results showed a significant difference between the two conditions, *t*(191) = −17.51, *p* < 0.001, indicating that participants assigned to the “justice sensitivity” condition experienced stronger feelings of personal injustice (*M* = 7.44, SD = 1.72) compared to those assigned to the “control” condition (*M* = 2.58, SD = 2.07).

To test the moderating role of (induced) justice sensitivity on the relationship between ambition and extreme behavior, we employed the same model of Study 1. Confirming the results of the first study, we found that ambition positively and significantly predicted extreme behavior [*b* = 0.43, (95% CI = 0.19, 0.67), *SE* = 0.121, *t* = 3.575, *p* < 0.001], while condition did not [*b* = −0.06, (95% CI = −0.24, 0.11), *SE* = 0.089, *t* = −0.705, *p* = 0.482]. Most importantly, we found a positive and significant effect of the interaction between condition and ambition on extreme behavior [*b* = 0.26, (95% CI = 0.02, 0.50), *SE* = 0.121, *t* = 2.117, *p* = 0.036], showing once again that the relationship between ambition and extreme behavior tended to be more strongly positive under “justice sensitivity” condition (Figure 3).^2^

**Figure 4:**
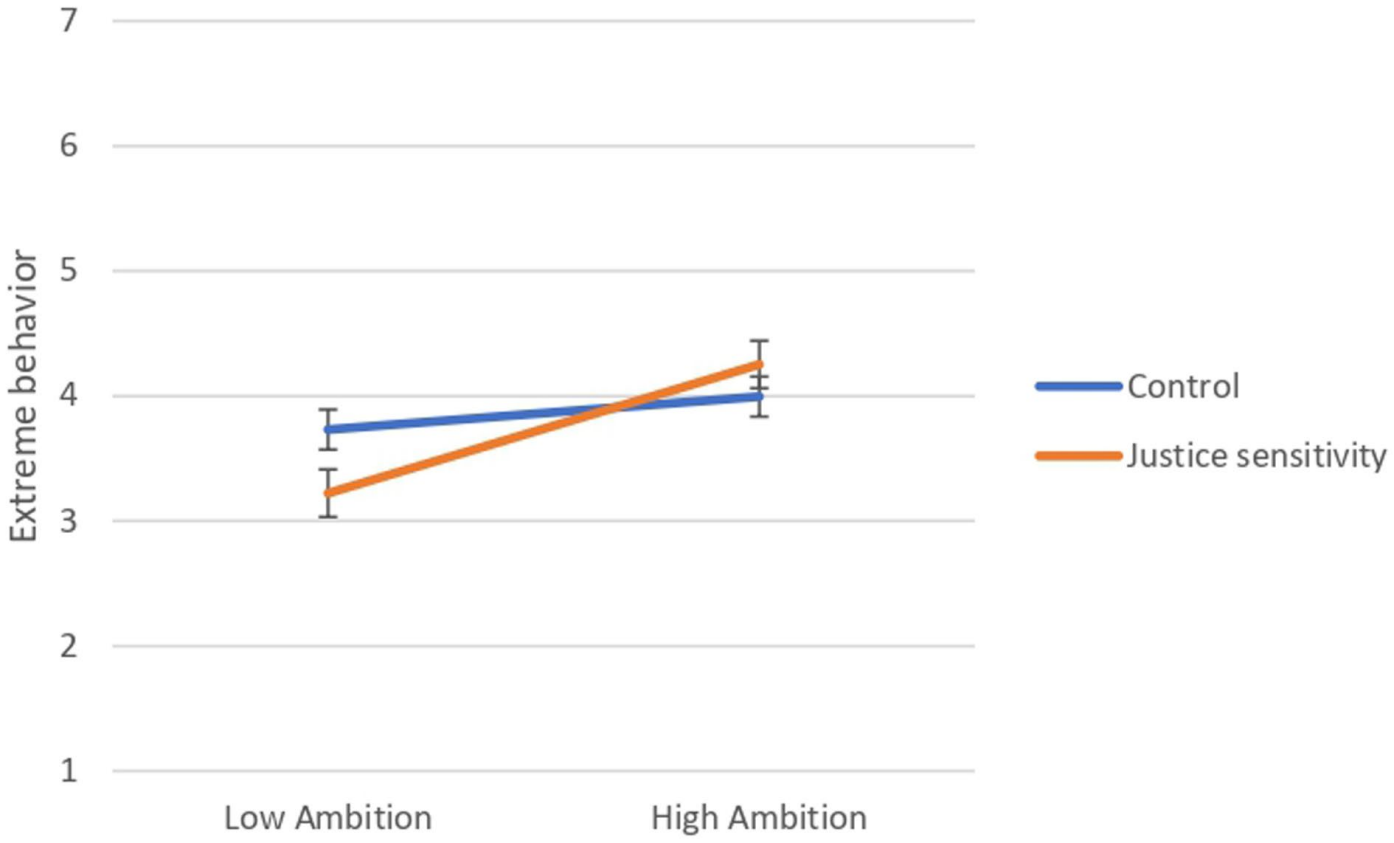
Results of simple-slope analysis to interpret the effect of the interaction between ambition and condition on extreme behavior (Study 2; *N* = 193).

The simple-slope analysis revealed that, akin to Study 1, the relationship between ambition and extreme behavior was positive and significant under the “justice sensitivity” condition, [*b* = 0.69, (95% CI = 0.32, 1.06), *SE* = 0.186, *t* = 3.713, *p* < 0.001], and it became non-significant under the “control” condition, [*b* = 0.18, (95% CI = −0.13, 0.48), *SE* = 0.156, *t* = 1.135, *p* = 0.258], (see Figure 4).

### 3.4. Discussion

The results obtained in Study 2 confirmed those of Study 1, using an experimental methodology and a sample from another nationality. As in the first study, we confirmed that the relationship between ambition and extreme behavior was moderated by relative deprivation. Ambitious individuals were significantly more likely to engage in extreme behavior in pursuit of a valued objective only after recalling three situations in which they felt they had been treated unfairly. After recalling three emotionally neutral situations, ambitious people were not significantly more likely to engage in extreme behavior in pursuit of a valued objective. Thus, ambitious people are more prone to extremism in reaction to personal injustice, rather than in reaction to everyday situations.

## 4. General discussion

In the two studies described presently we tested the moderating role of relative deprivation in the relationship between ambition and extreme behavior. Specifically, Study 1 employed an American sample and tested our hypothesis through a cross-sectional design, showing that ambitious individuals are prone to engage in extreme behavior when they experience negative feelings in reaction to relative deprivation (i.e., justice sensitivity). Moreover, we did not find an effect of justice sensitivity on extremism, in contrast to other findings that relative deprivation is “a key factor driving violent extremism across cultures and contexts” (Kunst and Obaidi, 2020, p. 55). Of course, the present study measured extremism and willingness to sacrifice generally and in pursuit of personally selected goals, rather than violent extremism in particular. In addition, it is important to note that literature distinguishes between egoistic (individual) and fraternal (group) relative deprivation (Runciman, 1966). Indeed, one can perceive that they are personally deprived (egoistic relative deprivation) or that a social group to which they belong is deprived (fraternal relative deprivation). Specifically, fraternal relative deprivation has been shown to be more closely related to protest than egoistic relative deprivation (Abeles, 1976), while egoistic relative deprivation has been shown to be more closely related to well-being than fraternal relative deprivation (Smith and Ortiz, 2002). The present study focused on egoistic relative deprivation, so it was perhaps foreseeable that justice sensitivity would not predict extreme behavior.

Due to the correlational nature of the data in Study 1, we were not able to draw causal inferences. Hence, we conducted a second study which tested the same hypothesis, employing an experimental design and an Italian sample. The results of Study 2 confirmed those of Study 1, demonstrating that ambitious individuals are likely to engage in extremism in reaction to personal injustice, but they are not significantly likely to engage in extremism in everyday situations. Akin to Study 1, we found a significant and positive effect of ambition on extremism, while we did not find any effect of assignment to the justice sensitivity (vs. control) condition on extremism.

Overall, our findings show that ambition predicts extreme behavior, supporting the results of the existing literature on this relationship (e.g., Resta et al., 2022a,c). Moreover, the present research extends the theoretical knowledge regarding the relationship between ambition and extreme behavior by identifying a particular condition under which this relationship is strengthened. Across the two studies, we predicted and found that negative reactions to relative deprivation, whether measured or induced, intensifies ambitious individuals’ likelihood to engage in extreme behavior in pursuit of valued causes or goals. Thus, the present findings enrich not only literature about ambition and extremism, but also that regarding relative deprivation.

The present study has several limitations. First, we used a self-report measure to assess extremism, rather than actual behavior, perhaps making the measure less realistic or subject to social desirability bias. This limitation was mitigated, however, by asking participants to indicate a goal very dear *to them.* Thus, we measured self-sacrifice in pursuit of a personal objective, enabling us to measure a realistic motivation to engage in extreme behavior rather than the motivation to engage in extremism for the sake of a general or imposed objective. A second limitation is that in both studies we measured rather than manipulated ambition. Future studies should address this issue by testing the effect of the interaction between ambition and justice sensitivity on extremism through an experimental design which manipulates both ambition and justice sensitivity.

Despite these limitations and the necessity of future research to address them, the present research yields fruitful practical implications. First, the present findings identified precise conditions under which ambitious individuals are more or less prone to engage in extreme behavior. Second, actions aimed to prevent extremism should focus on diminishing negative reactions to personal injustice, particularly in ambitious individuals. Ambitious people, indeed, may be served by modifying their reactions to situations of personal injustice. This could occur, for instance, through trainings aimed to prevent strong negative reactions that will lead them to the engagement in extreme behavior.

## Data availability statement

The raw data supporting the conclusions of this article will be made available by the authors, without undue reservation.

## Ethics statement

The studies involving human participants were reviewed and approved by the Ethics Committee of the Department of Developmental and Social Psychology at Sapienza University of Rome. The patients/participants provided their written informed consent to participate in this study.

## Author contributions

ER, AWK, and AP contributed to the study conception and design. Material preparation, data collection, and analysis were performed by ER and AP. The first draft of the manuscript was written by ER. Reviews and editing were provided by ME and all authors commented on previous versions of the manuscript. All authors read and approved the final manuscript.

## Funding

This work was supported by the research funding No AR22218161CD86A7 (“Progetti per Avvio alla Ricerca - Tipo 2”) awarded by Sapienza University of Rome to ER.

## Conflict of interest

The authors declare that the research was conducted in the absence of any commercial or financial relationships that could be construed as a potential conflict of interest.

## Publisher’s note

All claims expressed in this article are solely those of the authors and do not necessarily represent those of their affiliated organizations, or those of the publisher, the editors and the reviewers. Any product that may be evaluated in this article, or claim that may be made by its manufacturer, is not guaranteed or endorsed by the publisher.

## Footnotes

^1^To further test the effect of the predictors on extreme behavior we run a hierarchical regression that can be found in Supplementary material.

^2^To further test the effect of the predictors on extreme behavior we run a hierarchical regression that can be found in Supplementary material.

## References

[ref1] Abeles R. P. (1976). Relative deprivation, rising expectations, and black militancy. J. Soc. Issues 32, 119–137. doi: 10.1111/j.1540-4560.1976.tb02498.x

[ref2] Adam-Troian J. Bonetto E. Araujo M. Baidada O. Celebi E. Dono Martin M.. (2020). Positive associations between anomia and intentions to engage in political violence: cross-cultural evidence from four countries. Peace Conf. J. Peace Psychol. 26, 217–223. doi: 10.1037/pac0000385

[ref3] Bélanger J. (2013). The psychology of martyrdom. [Unpublished doctoral dissertation]. University of Maryland.

[ref4] Bélanger J. J. Caouette J. Sharvit K. Dugas M. (2014). The psychology of martyrdom: making the ultimate sacrifice in the name of a cause. J. Pers. Soc. Psychol. 107, 494–515. doi: 10.1037/a0036855, PMID: 25133728

[ref5] Belanger J. J. Kruglanski A. W. Kessels U. (2019). On sin and sacrifice: how intrinsic religiosity and sexual-guilt create support for martyrdom. Psychol. Res. Urban Soc. 2, 66–75. doi: 10.7454/proust.v2i2.40

[ref6] Bonebright C. A. Clay D. L. Ankenmann R. D. (2000). The relationship of workaholism conflict, life satisfaction, and purpose in life. J. Counsel. Pyshcol. 47, 469–477. doi: 10.1037/0022-0167.47.4.469

[ref7] Burke R. J. (1999). Workaholism and extra-work satisfactions. Int. J. Organ. Anal. 7, 352–364. doi: 10.1108/eb028906

[ref8] Callan M. J. Shead N. W. Olson J. M. (2011). Personal relative deprivation, delay discounting, and gambling. J. Pers. Soc. Psychol. 101, 955–973. doi: 10.1037/a0024778, PMID: 21875231

[ref9] Crosby F. (1976). A model of egotistical relative deprivation. Psychol. Rev. 83, 85–113. doi: 10.1037/0033-295X.83.2.85

[ref10] Dugas M. Bélanger J. J. Moyano M. Schumpe B. M. Kruglanski A. W. Gelfand M. J.. (2016). The quest for significance motivates self-sacrifice. Motiv. Sci. 2, 15–32. doi: 10.1037/mot0000030

[ref11] Ellemers N. (2002). “Social identity and relative deprivation” in Relative deprivation: Specification, development and integration. eds. Walker I. Smith H. J. (Cambridge: Cambridge University Press)

[ref12] Faul F. Erdfelder E. Buchner A. Lang A. G. (2009). Statistical power analyses using G* power 3.1: tests for correlation and regression analyses. Behav. Res. Methods 41, 1149–1160. doi: 10.3758/BRM.41.4.1149 19897823

[ref13] Grant P. R. (2008). The protest intentions of skilled immigrants with credentialing problems: a test of a model integrating relative deprivation theory with social identity theory. Br. J. Soc. Psychol. 47, 687–705. doi: 10.1348/014466607X269829 18166140

[ref14] Hayes A. F. (2018). Introduction to mediation, moderation, and conditional process analysis. New York: The Guilford Press.

[ref15] Kruglanski A. W. Bertelsen P. (2020). Life psychology and significance quest: a complementary approach to violent extremism and counter-radicalization. J. Pol. Intel. Count. Terror. 15, 1–22. doi: 10.1080/18335330.2020.1725098

[ref16] Kruglanski A. W. Molinario E. Jasko K. Webber D. Leander N. P. Pierro A. (2022). Significance quest theory. Perspect. Psychol. Sci. 17, 1050–1071. doi: 10.1177/17456916211034825, PMID: 35133911

[ref17] Kruglanski A. W. Szumowska E. Kopetz C. H. Vallerand R. J. Pierro A. (2021). On the psychology of extremism: how motivational imbalance breeds intemperance. Psychol. Rev. 128, 264–289. doi: 10.1037/rev0000260, PMID: 32915010

[ref18] Kunst J. R. Obaidi M. (2020). Understanding violent extremism in the 21st century: the (re) emerging role of relative deprivation. Curr. Opin. Psychol. 35, 55–59. doi: 10.1016/j.copsyc.2020.03.010, PMID: 32344297

[ref19] Machlowitz M. (1980). Workaholics: Living with them, working with them. Boston: Addison-Wesley Publishing Company.

[ref20] Meuleman B. Abts K. Schmidt P. Pettigrew T. F. Davidov E. (2020). Economic conditions, group relative deprivation and ethnic threat perceptions: a cross-national perspective. J. Ethn. Migr. Stud. 46, 593–611. doi: 10.1080/1369183X.2018.1550157

[ref21] Ng T. W. H. Sorensen K. L. Feldman D. C. (2007). Dimensions, antecedents, and consequences of Workaholism: a conceptual integration and extension. J. Organ. Behav. 28, 111–136. doi: 10.1002/job.424

[ref22] Ohno H. Lee K. T. Maeno T. (2023). Feelings of personal relative deprivation and subjective well-being in Japan. Behav. Sci. 13, 158–176. doi: 10.3390/bs13020158, PMID: 36829387 PMC9952549

[ref23] Resta E. Ellenberg M. Kruglanski A. W. Pierro A. (2022a). Marie curie vs. Serena Williams: ambition leads to extremism through obsessive (but not harmonious) passion. Motiv. Emot. 46, 382–393. doi: 10.1007/s11031-022-09936-3

[ref24] Resta E. Ellenberg M. Kruglanski A. W. Pierro A. (2022b). The ambition scale: Italian and English validation. [Unpublished Manuscript].

[ref25] Resta E. Kruglanski A. W. Ellenberg M. Pierro A. (2022c). Ambition-driven aggression in response to significance-threatening frustration. [Unpublished Manuscript].

[ref26] Robinson B. E. Flowers C. Carroll J. J. (2001). Work stress and marriage: a theoretical model examining the relationships between workaholism and marital cohesion. Int. J. Stress. Manag. 8, 165–175. doi: 10.1023/A:1009533415030

[ref27] Runciman W. G. (1966). Relative deprivation and social justice. London: Routledge.

[ref28] Schmitt M. Baumert A. Gollwitzer M. Maes J. (2010). The justice sensitivity inventory: factorial validity, location in the personality facet space, demographic pattern, and normative data. Soc. Justice Res 23, 211–238. doi: 10.1007/s11211-010-0115-2

[ref29] Schreurs B. Hamstra M. R. Jawahar I. M. Akkermans J. (2021). Perceived overqualification and counterproductive work behavior: testing the mediating role of relative deprivation and the moderating role of ambition. Pers. Rev. 50, 1038–1055. doi: 10.1108/PR-05-2019-0237

[ref30] Smith H. J. Ortiz D. J. (2002). “Is it just me?” in Relative deprivation. eds. Walker I. Smith H. (Cambridge: Cambridge University Press)

[ref31] Stark O. Taylor J. E. (1991). Migration incentives, migration types: the role of relative deprivation. Econ. J. 101, 1163–1178. doi: 10.2307/2234433

[ref32] Stiles B. L. Liu X. Kaplan H. B. (2000). Relative deprivation and deviant adaptations: the mediating effects of negative self-feelings. J. Res. Crime Delinq. 37, 64–90. doi: 10.1177/0022427800037001003

[ref33] Stouffer S. A. Suchman E. A. DeVinney L. C. Star S. A. Williams R. M. (1949). The American soldier: Adjustment during army life. New Jersey: Princeton University Press.

[ref34] Van den Bos K. Van Veldhuizen T. S. Au A. K. (2015). Counter cross-cultural priming and relative deprivation: the role of individualism–collectivism. Soc. Justice Res 28, 52–75. doi: 10.1007/s11211-014-0230-6

[ref35] Webber D. Chernikova M. Kruglanski A. W. Gelfand M. J. Hettiarachchi M. Gunaratna R.. (2018). Deradicalizing detained terrorists. Polit. Psychol. 39, 539–556. doi: 10.1111/pops.12428

[ref36] Zoogah D. B. (2010). Why should I be left behind? Employees’ perceived relative deprivation and participation in development activities. J. Appl. Psychol. 95, 159–173. doi: 10.1037/a0018019, PMID: 20085413

