# Supplementary material for: Ambition and extreme behavior: relative deprivation leads ambitious individuals to self-sacrifice
Source: Front Psychol. 2023 Jul 12;14:1108006. doi: 10.3389/fpsyg.2023.1108006 (PMC10370493; doi:10.3389/fpsyg.2023.1108006)
Supplement: Supplementary file 1 [file Data_Sheet_1.docx]

**Supplemental Materials**

**Jusitice Sensitivity Scale**

Please indicate how much you agree with each of the following items, using the following scale:

1= Definitely disagree – 5 = Definitely agree

| Items |  |
| --- | --- |
| 1. I am bothered when people get what I deserve. | 1 2 3 4 5 |
| 2. I become demoralized when I have less opportunity to improve my abilities. | 1 2 3 4 5 |
| 3. I am bothered when I have to work hard to get something others get easily. | 1 2 3 4 5 |
| 4. I am irritated when I have less opportunity to realize my ambitions. | 1 2 3 4 5 |
| 5. I get frustrated when I bump into more obstacles than others to reach my goals. | 1 2 3 4 5 |

**Ambition Scale**

Please indicate how much you agree with each of the following items, using the following scale:

1= Definitely disagree – 5 = Definitely agree

| Items |  |
| --- | --- |
| 1. I am ambitious. | 1 2 3 4 5 |
| 2. One of my goals is doing something that leaves a mark. | 1 2 3 4 5 |
| 3. I aim to succeed. | 1 2 3 4 5 |
| 4. I aspire to do something special. | 1 2 3 4 5 |
| 5. I never stop trying to overcome my limits. | 1 2 3 4 5 |
| 6. I always aim higher than I know I can do. | 1 2 3 4 5 |
| 7. I always try to stand out in what I do. | 1 2 3 4 5 |
| 8. I aim to do or have something enviable. | 1 2 3 4 5 |
| 9. I aim to hold positions of prestige and responsibility. | 1 2 3 4 5 |
| 10. Attaining recognition, respect and consideration for what I do is very important to me. | 1 2 3 4 5 |

**Extreme Behavior**

Now think about a cause that is very important for you.

Please list the cause below:_______________

Indicate your agreement with each of the following statements.

Scale: 1= Do not agree at all – 7 = Very strongly agree

| Items |  |
| --- | --- |
| 1. It is senseless to sacrifice one’s life for a cause. | 1 2 3 4 5 6 7 |
| 2. I would defend a cause to which I am truly committed even if my loved ones rejected me. | 1 2 3 4 5 6 7 |
| 3. I would be prepared to endure intense suffering if it meant defending an important cause. | 1 2 3 4 5 6 7 |
| 4. I would be ready to give my life for a cause that is extremely dear to me. | 1 2 3 4 5 6 7 |
| 5. I would be willing to give away all my belongings to support an important cause. | 1 2 3 4 5 6 7 |

**Manipulation of Justice Sensitivity**

1. Please describe a time when you felt bothered because you had fewer opportunities than others to fulfill your ambitions:

____________________________________________________________________________________________________________________________________________________________________________________________________________________________________________________________________________________

1. Please describe a time when you felt frustrated because you had to work hard to achieve a goal that others easily achieved:

____________________________________________________________________________________________________________________________________________________________________________________________________________________________________________________________________________________

1. Please describe a situation where you felt bothered because others got what you deserved:

____________________________________________________________________________________________________________________________________________________________________________________________________________________________________________________________________________________

**Control Condition**

1. Please carefully describe your last meal:

____________________________________________________________________________________________________________________________________________________________________________________________________________________________________________________________________________________

1. Please carefully describe the prototypical restaurant you are used to going to:

____________________________________________________________________________________________________________________________________________________________________________________________________________________________________________________________________________________

1. Please carefully describe your typical day:

____________________________________________________________________________________________________________________________________________________________________________________________________________________________________________________________________________________

**Further Analyses for Study 1**

In order to test the effect of our predictors on extreme behavior, without controlling for each other, we run a hierarchical regression. Specifically, we entered ambition (Model 1), justice sensitivity (Model 2), and the interaction (Model 3) as predictors and extreme behavior as criteria. Results are reported in the following table.

| Predictors | β | *SE* | | *t* | LL CI | UL CI | r_partial_ | VIF | *R*^2^ | ∆*R*^2^ |
| --- | --- | --- | --- | --- | --- | --- | --- | --- | --- | --- |
| Model 1 |  |  | |  |  |  |  |  | .03 | .03 |
| Ambition | .18 | .07 | 3.22** | | .09 | .36 | .18 | 1 |  |  |
| Model 2 |  |  |  | |  |  |  |  | .03 | .004 |
| Ambition | .18 | .07 | 3.04** | | .07 | .35 | .17 | 1.02 |  |  |
| Justice sensitivity | .06 | .07 | 1.05 | | -.06 | .21 | .06 | 1.02 |  |  |
| Model 3 |  |  |  | |  |  |  |  | .05 | .02 |
| Ambition | .18 | .07 | | 3.14** | .08 | .35 | .18 | 1.02 |  |  |
| Justice sensitivity | .04 | .07 | | .75 | -.08 | .19 | .04 | 1.04 |  |  |
| Ambition*Justice sensitivity | .14 | .06 | | 2.45* | .03 | .27 | .14 | 1.02 |  |  |

**p* < .05. ***p* < .01

**Further Analyses for Study 2**

In order to test the effect of our predictors on extreme behavior without controlling for each other, we run a hierarchical regression. Specifically, we entered ambition (Model 1), condition (Model 2), and the interaction (Model 3) as predictors and extreme behavior as criteria. Results are reported in the following table.

| Predictors | β | *SE* | | *t* | LL CI | UL CI | r_partial_ | VIF | *R*^2^ | ∆*R*^2^ |
| --- | --- | --- | --- | --- | --- | --- | --- | --- | --- | --- |
| Model 1 |  |  | |  |  |  |  |  | .05 | .05 |
| Ambition | .23 | .09 | 3.18** | | .11 | .46 | .22 | 1 |  |  |
| Model 2 |  |  |  | |  |  |  |  | .04 | .002 |
| Ambition | .23 | .09 | 3.23** | | .11 | .47 | .23 | 1.03 |  |  |
| Condition | -.04 | .09 | -.60 | | -.23 | .12 | -.04 | 1.03 |  |  |
| Model 3 |  |  |  | |  |  |  |  | .06 | .02 |
| Ambition | .26 | .09 | | 3.57** | .15 | .50 | .25 | 1.06 |  |  |
| Condition | -.05 | .09 | | -.71 | -.24 | .11 | -.05 | 1.03 |  |  |
| Ambition*Condition | .15 | .09 | | 2.18* | .01 | .37 | .15 | 1.03 |  |  |

**p* < .05. ***p* < .01
